# A new approach to improving undergraduate research

**DOI:** 10.5116/ijme.5749.6b73

**Published:** 2016-06-11

**Authors:** Saleh K. Alqaryan

**Affiliations:** 1 Qassim University, College of Medicine, Saudi Arabia

**Keywords:** Improving undergraduate research, Saudi Arabia

## Introduction

Research is the keystone in modern-day evidence-based medical practice, the importance of which cannot be overemphasized. Many students sink into the depths of despair when faced with the prospect of undertaking research. In developing countries, the opportunity to engage in research from early in one’s career is almost non-existent. In Saudi Arabia, for example, one study found that there are 23 researchers for every 100,000 population. By contrast, this statistic amounts to 500 researchers per 100,000 population in developed countries.^1^

This presents a significant challenge not only here in Saudi Arabia but also around the globe. Therefore, I propose a framework of dynamic tools that aims to change medical students’ perceptions of academic research and offer them more productive and successful research opportunities by utilizing the “practice makes perfect” model and taking research out of the office and bringing it online. This will result in more constructive, time-saving, and collaborative research. Moreover, this platform will allow medical students on a global level to come together to form a diverse community of researchers who will, in turn, enrich each other’s skills and widen their horizons.

The project is based on a system of mentorship to provide guidance and feedback to those who wish to enhance their research careers. The project begins by dividing the participants into two groups: the mentors and the mentees. Mentors will include medical faculty, consultants, and specialists. Mentees will include medical students, interns, and residents. The research foci will be categorized as active (currently conducting the study), inactive (stopped), and finished (findings have been published and presented).

By creating lists of specialties, along with their affiliated mentors, each mentor will be given a profile that includes their qualifications, publications, contact information, research interests, and indicate the number of foci to which he/she is assigned that are active, inactive, or finished. One method to commence work on a study is by the mentee determining a research focus and sending proposals to possible mentors in the appropriate fields. The mentors will then have the option of accepting or rejecting the proposal. The other method is by a mentor creating a research focus on which the mentees can apply to work. The mentor can then review the applicants’ profiles and choose the candidate that is best suited to work on the study. Upon activation of the research foci, the mentors provide the objectives of the studies, assign tasks to all the mentees, and sets a timeframe for completion. Inactive foci can be reactivated through collaboration with other interested mentors. Before changing the category of the research focus from “active” to “finished,” a survey will be distributed among the focus members to collect their feedback on the experience and allow them to rate their mentor.

It is imperative to recognize that this project is designed to suit research undertaken in certain fields of study and not others. Concerns regarding both confidentiality and professionalism are valid and are anticipated limitations of this project. Certain rules and regulations must be established to resolve these problems. Despite that, this project will create a unique opportunity for collaborative research that is not subject to a significant amount of bureaucracy and allows for greater autonomy than traditional research projects.

### Conflict of Interest

The author declares that there are no conflicts of interest.
